# A new global and comprehensive model for ICU ventilator performances evaluation

**DOI:** 10.1186/s13613-017-0285-2

**Published:** 2017-06-17

**Authors:** Nicolas S. Marjanovic, Agathe De Simone, Guillaume Jegou, Erwan L’Her

**Affiliations:** 1 Urgences Adultes/SAMU 86, CHU de Poitiers, 86000 Poitiers Cedex, France; 2 ABS-Lab, Laboratoire d’Anatomie, Biomécanique et Simulation, Université de Poitiers, Rue de la Milétrie, 86000 Poitiers Cedex, France; 3 B-Com Technical Research Institute, 29200 Brest Cedex, France; 4 CeSim/LaTIM INSERM UMR 1101, Université de Bretagne Occidentale, Rue Camille Desmoulins, 29200 Brest Cedex, France; 5 Médecine Intensive et Réanimation, CHRU de Brest, Boulevard Tanguy Prigent, 29200 Brest Cedex, France

## Abstract

**Background:**

This study aimed to provide a new global and comprehensive evaluation of recent ICU ventilators taking into account both technical performances and ergonomics.

**Methods:**

Six recent ICU ventilators were evaluated. Technical performances were assessed under two FIO_2_ levels (100%, 50%), three respiratory mechanics combinations (*Normal*: compliance [*C*] = 70 mL cmH_2_O^−1^/resistance [*R*] = 5 cmH_2_O L^−1^ s^−1^; *Restrictive*: *C* = 30/*R* = 10; *Obstructive*: *C* = 120/*R* = 20), four exponential levels of leaks (from 0 to 12.5 L min^−1^) and three levels of inspiratory effort (P0.1 = 2, 4 and 8 cmH_2_O), using an automated test lung. Ergonomics were evaluated by 20 ICU physicians using a global and comprehensive model involving physiological response to stress measurements (heart rate, respiratory rate, tidal volume variability and eye tracking), psycho-cognitive scales (SUS and NASA-TLX) and objective tasks completion.

**Results:**

Few differences in terms of technical performance were observed between devices. Non-invasive ventilation modes had a huge influence on asynchrony occurrence. Using our global model, either objective tasks completion, psycho-cognitive scales and/or physiological measurements were able to depict significant differences in terms of devices’ usability. The level of failure that was observed with some devices depicted the lack of adaptation of device’s development to end users’ requests.

**Conclusions:**

Despite similar technical performance, some ICU ventilators exhibit low ergonomics performance and a high risk of misusage.

**Electronic supplementary material:**

The online version of this article (doi:10.1186/s13613-017-0285-2) contains supplementary material, which is available to authorized users.

## Background

Mechanical ventilation is a fundamental part of critical care, and the accuracy of ventilatory settings is of utmost importance. When dealing with unstable patients, a bad technological performance may cause a patient harm, while low tidal volume (VT) and high positive expiratory pressure (PEEP) are key points for protective ventilation [1, 2]. Ineffective effort and asynchrony correction [3], along with effective triggering [4], may decrease inspiratory work and improve patients’ outcome. Bench-test studies are essential to assess the technical characteristics of ventilators and determine their efficiency during critical care [5–9].

Besides technical performance, another major aspect of a device’s reliability is its usability. Usability is defined as the extent to which a device can be used by specified users to achieve specific goals effectively, efficiently and satisfactorily, in a specified context of use. Usability is mainly related to the quality of the human–machine interface. Improved interface seems mandatory to limit human errors that could exacerbate morbidity and mortality [10–12]. There are few studies dedicated to ventilator ergonomics evaluation, and those that do exist are often limited to timed task and/or easy user-friendliness assessments [13–16].

**Fig. 1:**
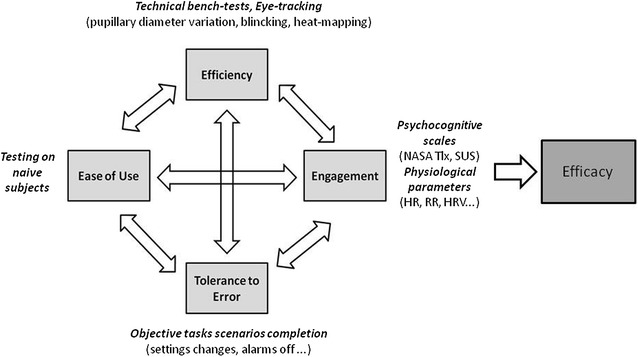
The concept of devices’ global evaluation. In order to assess ‘*efficacy*’ of a device, we considered that a global ergonomics evaluation required evaluating concomitantly ‘*efficiency*’, ‘*engagement*’, ‘*ease of use*’ and ‘*tolerance to error*’, as these four dimensions may be considered as interdependent. Tolerance to error was evaluated through the objective tasks completion scenarios. While considering that an easy-to-use device should be easily managed by a skilled physician, but not familiar to that specific device, we took particular attention to included naive subjects in the evaluation. Bench testings do explore the most important technical determinants of the efficiency of a device (tidal volume accuracy, triggering, etc.). Efficiency assessment might also include interfaces’ performance evaluation. For such sake, we used pupillary diameter variation which can be considered as a determinant of stress. Other eye-tracking tools such as blinking measurements or heat mapping may also have been used. Engagement during use of the device was evaluated through the use of psycho-cognitive scales, combined with physiological parameters measurements. Heart rate variability (HRV) may also have been measured for such sake

The aims of this study were to provide a new global and comprehensive evaluation of recent ICU ventilators, taking into account both their technical performance and a comprehensive ergonomics evaluation (Fig. 1).

## Methods

### Tested devices

Six ICU ventilators were evaluated in a dedicated bench test. All ventilators were provided free of charge by manufacturers: (1) Dräger V500 (Lubeck, Germany); (2) Covidien PB980 (Mansfield, MA, USA); (3) Philips V680 (Murrysville, PA, USA); (4) Hamilton S1 (Bonaduz, Switzerland); (5) General Electrics R860 (Fairfield, CT, USA); and (6) Maquet Servo-U (Göteborg, Sweden). General characteristics of the devices are provided in the online repository (Additional file 1: Table S1).

### Technical performance

Measurements were taken using an ASL5000™ lung simulator (Ingmar, Pittsburgh, PA, USA) under constant room temperature (22 °C), simulator temperature (37 °C), under dry ambient pressure (ATPD) conditions, and converted into body temperature and pressure, saturated (BTPS) as previously described [6, 17]. Technical performances were assessed under two FIO_2_ levels (100%, 50%), three respiratory mechanics combinations (*Normal*: resistance [*R*] = 5 cmH_2_O L^−1^ s^−1^; compliance [*C*] = 70 mL cmH_2_O^−1^, *Restrictive*: *R* = 10 cmH_2_O L^−1^ s^−1^; C = 30 mL cmH_2_O^−1^ and *Obstructive*: *R* = 20 cmH_2_O L^−1^ s^−1^; C = 120 mL cmH_2_O^−1^), three exponential levels of leaks (L1 = 3.5–4.0 L min^−1^; L2 = 5.0–7.0 L min^−1^; L3 = 9.0–12.5 L min^−1^) and three levels of inspiratory effort (P0.1 = 2, 4 and 8 cmH_2_O) in volume-controlled continuous mandatory ventilation (VC-CMV) and pressure-controlled continuous spontaneous ventilation (PC-CSV) [4]. Triggering capabilities, volume and pressurization accuracy were evaluated under the different respiratory mechanics at standardized respiratory settings. The asynchrony index [18] was defined as the number of asynchrony events divided by the total respiratory rate and expressed in percentage [3]. Asynchronies were evaluated under the different respiratory mechanics and the three exponential levels of leaks and inspiratory efforts.

Error was evaluated as the average difference between set and true dimension value (VT, PEEP, pressure support). Accuracy was a priori considered for an error value below 10% for all parameters. Precision of the dimension was defined as the range value of the dimension, considering that a narrow range was the more precise. An asynchrony index greater than 10% was also considered clinically significant [3, 19].

Details about the technical performance evaluation are provided in the online repository.

### Ergonomics

For ergonomics evaluation, we included, as a reference, the use of a device that was familiar to all physicians (Avea, Carefusion, San Diego, CA, USA).

#### ICU physicians involved in ergonomics evaluation

Twenty senior ICU physicians from five different ICUs were included in the evaluation. Each physician tested 3–4 devices in a randomized order; each device was tested 11 or 12 times. All physicians used the Avea in their daily clinical practice; we paid particular attention to the fact that none of them were familiar with the tested devices (naive subjects), even though some of them were in some cases familiar with other devices from the same manufacturer (see Additional file 1: Table S2, Additional file 1: Table S3).

#### Objective task completion

The ICU physicians had to complete 11 specific tasks for each ventilator, four mainly dedicated to monitoring and seven to setting: (a) alarm control (users must shut down alarms, identify the reason and modify setting to stop alarms); (b) mode recognition (exact reading of the ventilator mode set by investigator); (c) identify humidification system on the screen and modify it; (d) ventilator setting reading (VT, ventilation rate, PEEP and trigger value); (e) power on the ventilator; (f) start ventilation; (g) set inspiratory flow to a value defined by the investigator (40–80 L min^−1^); (h) ventilator mode modification; (i) set cycling to 60%; (j) non-invasive ventilation mode activation; and (k) ventilator extinction (complete ventilator powering down). In each group of tests (i.e. monitoring or setting), tasks were to be performed in a randomized order. The test was a priori considered as a failure if the correct response was given after more than 120 s, or if the physicians did not provide a correct response or abandoned the task. Due to technical constraints, we chose not to use a high-fidelity environment with a manikin, but to perform measurements with the ventilators connected solely to the test lung. Besides a task failure rate evaluation, these scenarios were also dedicated to enable usability and mental workload scoring using psycho-cognitive scales.

#### Psycho-cognitive scales evaluation

Psycho-cognitive scorings were performed immediately after all objective tasks completion.

##### System Usability Scale (SUS)

The SUS is a reliability tool, developed to measure a device’s usability [20]. It consists of a ten-item questionnaire and assesses usability by different aspects: effectiveness (ability of users to complete tasks); efficiency (level of resource used in performing tasks); and satisfaction (subjective reactions to using the system). The SUS score has a range of 0–100, the highest score being the best value (‘simple to use’).

##### Mental workload evaluation using the NASA-TLX

Mental workload is a subjective ergonomic measurement and an indicator for interface development, assessment and comparison. NASA-TLX is a multidimensional tool that was developed by the National Aeronautics and Space Administration’s Ames Research Center in 1986 for perceptual mental workload evaluations using the Task Load Index measurement through three dimensions, dependent on the user’s perception of the task (mental workload, temporal workload and physical workload) and three dimensions dependent on the interaction between the subject and the task itself, which may be mostly related to the interface (effort, performance and frustration). Each dimension is rated using a Likert-type scale ranging from 0 to 100. The second part of Task Load Index calculation intends to create an individual weighting of these dimensions by letting the subjects compare them pairwise, based on their perceived importance. These 15 comparison pairings thus enable the inter-/intra-individual variability of the overall score to decrease. The overall workload score for each subject is composed by multiplying each rating by the weight given to that factor by that subject. The sum of the weighted ratings for each task is divided by 15 (the sum of the weights). The higher the Task Load Index, the higher the mental workload and the more difficult it is to use the device.

#### Physiological measurements

Several physiological parameters were recorded during the completion of the objective tasks. Pupil diameter modifications were assessed using an eye-tracking system (SMI ETG 1, SensoMotoric Instruments GmbH, Teltow, Germany) (Additional file 1: Fig. S1); heart and respiratory rate and thoracic volume variations were measured using a biometric belt (Hexoskin, Montréal, Canada). Analysis consisted of a data treatment by a systems’ activation count, which corresponded to highly different values, as compared to baseline. Each of these activations is numerically integrated in order to evaluate the number of physiological variations in response to tasks. These activations are considered to be adequate stress indicators.

#### The concept of global ergonomics evaluation

A global and comprehensive model for a device’s efficacy evaluation needs to either assess technical performances and ergonomics or thus to explore four different dimensions (Fig. 1). Each of these four dimensions can be explored separately, but they are all related one to the other. Tolerance to error was evaluated through the objective tasks completion scenarios. While considering that an easy-to-use device should be easily managed by a skilled physician, but not familiar to that specific device, we took particular attention to included naive subjects in the evaluation. Bench testings do explore the most important technical determinants of the efficiency of a device (tidal volume accuracy, triggering, etc.). Efficiency evaluation might also include interface’s performance evaluation. For such sake, we used pupillary diameter variation which can be considered as a determinant of stress. Engagement during use of the device was evaluated through the use of psycho-cognitive scales, combined with physiological parameters measurements.

### Statistical analysis

Parameters were calculated over 10–20 cycles after signal stabilization and are provided as mean ± SD to calculate error and as median ± interquartile to evaluate precision of the dimension, in response to respiratory mechanics changes. Data were compared using analysis of variance (ANOVA) and nonparametric Friedman and Wilcoxon signed-rank test. A *p* value <0.05 was considered statistically significant. Statistical analysis was performed using MedCalc 12.7.4 for Windows (MedCalc software, Ostend, Belgium).

## Results

### Technical performances

#### Tidal volume accuracy (Fig. 2)

**Fig. 2:**
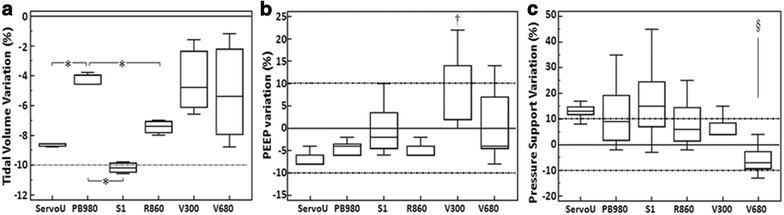
*Box plot* of tidal volume (VT) (**a**), positive end-expiratory pressure (PEEP) (**b**) and pressure support (PS) (**c**). *Dotted lines* represent the 10% error range. *Black line* represents exact VT value delivery. Values are provided as median and interquartile. A *p* value equal or below 0.05 was considered significant. *p < 0.05; ^§^ *p* < 0.05 as compared to S1, PB980 and Servo-U. ^†^ *p* < 0.005 as compared to R860, PB980 and Servo-U. There was a significant difference in terms of VT delivery precision between devices (**a**; *p* = 0.0498). All devices except S1 depicted a median VT value within the 10% error range. PB980 had the lowest error in terms of VT delivery, but Servo-U had the highest precision in response to respiratory mechanics modifications. V500 and V680 had relatively low error, but low precision in response to respiratory mechanics modifications. While median PEEP delivery was within the reliability range for all devices (**b**), V500 was significantly different to the other devices in terms of precision. S1 had the lowest error, but with rather low precision. Servo-U, PB980 and R860 had low error and high precision. When considering mean values, three devices delivered PS values higher than the reliability range (Servo-U: 13 ± 6%; PB980: 11 ± 12%; S1: 17 ± 14.4%; *p* < 0.001). Pressure support delivery was higher than the 10% error range for two devices (Servo-U and S1). Precision in response to respiratory mechanics modifications was low for PB980, S1 and R860

There was a significant difference in terms of tidal volume delivery precision between devices (Fig. 2a; *p* = 0.0498). All devices except S1 depicted a median tidal volume value within the 10% error range (VT = 449 ± 2 mL; ΔDVT = −10.2%). PB980 had the lowest error in terms of tidal volume delivery, but Servo-U had the higher precision in response to respiratory mechanics modifications. V500 and V680 had relatively low error, but low precision in response to respiratory mechanics modifications.

#### Pressurization accuracy (Fig. 2)

Pressurization accuracy differed between devices. Mean PEEP accuracy was similar between devices, except for V500 in the obstructive condition (pressurization error = 18 ± 5%). Three ventilators delivered a mean pressure support over the 10% error range (Servo-U: 13 ± 6%; PB980: 11 ± 12%; S1: 17 ± 14.4%; *p* < 0.001). S1 had the lowest error, but with rather low precision. Servo-U, PB980 and R860 had low error and high precision. V500 and V680 had low precision.

#### Triggering evaluation (Fig. 3)

No differences were observed between devices in terms of inspiratory triggering. Triggering delay was below 150 ms among the different respiratory mechanics, but exceeded 200 ms in obstructive conditions, except for PB980. Triggering pressure presented a large difference among the devices (*p* = 0.0004) and was higher for S1 (*p* = 0.0003) and R860 (*p* = 0.001).

**Fig. 3:**
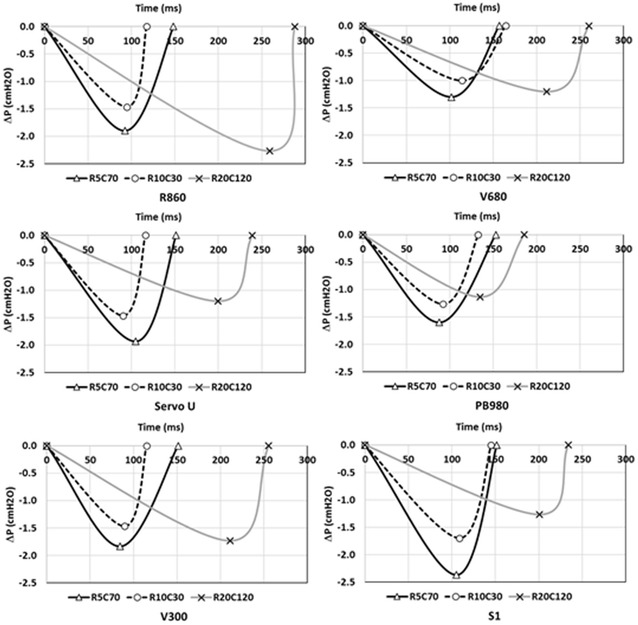
Triggering evaluation according to respiratory mechanics combinations. R: resistance; C: compliance; ΔP: maximal pressure drop required to trigger inspiration; DT: triggering delay, from the onset of the airway pressure decay (beginning of the patient’s effort) to flow delivery (beginning of ventilator pressurization); DP: pressurization delay, from the airway pressure signal rise to a return to positive pressure; DI: overall inspiratory delay (DT + DP). *Triangle* and *black line*: results for the ‘Normal’ respiratory mechanics; *circle* and *dotted line*: results for the ‘Restrictive’ respiratory mechanics; *cross* and *grey line*: results for the ‘Obstructive’ respiratory mechanics (*Normal*: resistance [*R*] = 5 cmH_2_O L^−1^ s^−1^; Compliance [*C*] = 70 mL cmH_2_O^−1^, *Restrictive*: R = 10 cmH_2_O L^−1^ s^−1^; C = 30 mL cmH_2_O^−1^ and *Obstructive*: R = 20 cmH_2_O L^−1^ s^−1^; C = 120 mL cmH_2_O^−1^). The *figure* presents individual results for each ventilator at the different respiratory mechanics combinations. *First point of each curve* represents inspiratory effort initiation; *second point* represents maximal depressurization (ΔP) before inspiratory pressure increase. There was no significant difference in terms of DT and DI between ventilators, nor in terms of maximal depressurization (ΔP). DI variability according to respiratory mechanics was higher for V500 and R860

#### Asynchrony management (Fig. 4)

Mean asynchrony indexes were equal to 31 and 14.5% under standard or non-invasive ventilatory modes, respectively, for all devices. All ventilators presented an asynchrony index of over 10% without using the non-invasive ventilation mode. While using non-invasive ventilation algorithms, the asynchrony index was lower for R860 and Servo-U (9.6%) as compared to V500 (14.6%), V680 (17.5%) and PB980 (18.3%; *p* < 0.05) (Additional file 1: Fig. S2). Most frequent asynchronies were prolonged cycles and ineffective efforts, ineffective efforts being most of the time associated with prolonged cycles.

**Fig. 4:**
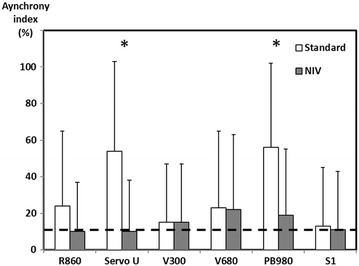
Asynchrony index with or without the non-invasive ventilation mode. The asynchrony index (AI) is presented as mean ± SD and was measured under three different levels of exponential leaks (L1 = 3.5–4.0 L min^−1^; L2 = 5.0–7.0 L min^−1^; L3 = 9.0–12.5 L min^−1^) and three levels of inspiratory effort (P0.1 = 2, 4, 8 cmH_2_O). ‘Standard’ represents measurements performed under PC-CSV using unmodified manufacturers’ settings in terms of inspiratory and expiratory triggering; ‘NIV’ represents the measurements performed under the same conditions, while switching the ventilator to the NIV mode. *Dotted line* represents the 10% AI clinical level of significance. *p value <0.001. All ventilators presented an AI over 10% without NIV mode (‘standard’ invasive PC-CSV setting). Under the same leaks conditions, switching the ventilator to the NIV mode enabled a decrease in the AI to below a 10% value for the R860 and Servo-U. V500 and S1 measurements did not depict a significant impact of the NIV mode, while ‘standard’ settings provided rather satisfactory results in terms of leak management

### Ergonomics evaluation

#### Objective task completion (Table 1)

Of all ventilators, our reference device the Avea had the best success rate. In our comparison of the six ventilators, Covidien PB980 had the best results and the Servo-U the worst. A minority of users could power on Servo-U, but always took longer than the predefined 120 s time range. The V500 was the fastest ventilator to power on. Only 36% of the ICU physicians were able to power on Servo-U and always with over a 1-min delay. No users could set the inspiratory flow on Servo-U, and only 18% of them succeeded in the same task with S1. Difficulties in activating the non-invasive ventilation mode were frequent with Servo-U, V500 and V680. Servo-U had the worst global results (tasks failure rate = 42%) compared to our reference (Avea; tasks failure rate = 13%) (*p* = 0.12). The lack of sensitivity of the S1 touch screen proved to be a barrier for some task completion and a significant source of confusion. The settings reading represented one of the most difficult tasks, whatever the device. Sensitivity analysis while deleting powering on and switching off tasks did not significantly modify the overall results.

**Table 1:** Objective tasks completion rate

Device	Power on (%)	Start ventilation (%)	Inspiratory flow setting (%)	Ventilatory mode modification (%)	Cycling setting (%)	NIV mode activation (%)
Carefusion Avea	92	100	100	100	75	75
Dräger V500	100	92	100	100	92	50
Covidien PB980	100	100	100	91	100	100
Philips V680	100	100	50^§^	91	91	40^†^
Hamilton S1	91	91	18^§^	82	91	91
GE R860	100	100	100	91	100	91
Maquet Servo-U	36*	100	0^¤^	100	45^‡^	27^†^

Device	Ventilator offset (%)	Alarm shut down (%)	Mode recognition (%)	Humidification system recognition (%)	Settings reading (%)	Overall
Carefusion Avea	100	100	100	75	42	87%/85%
Dräger V500	100	75	100	42	25	80%/75%
Covidien PB980	73*	64	100	73	45	86%/86%
Philips V680	100	82	100	55	18	75%/70%
Hamilton S1	100	91	100	36	27	74%/70%
GE R860	100	64	100	9^‖^	27	80%/76%
Maquet Servo-U	100	82	100	0^‖^	45	58%^#^/55%^‖^

Results are presented as the different objective tasks success rate, expressed as the percentage of successful attempts. ICU’s physicians with mechanical ventilation’s knowledge had to complete 11 specific tasks of variable clinical importance for each ventilator, 4 mainly dedicated to monitoring and 7 to setting. Overall provides results of the entire bunch of test (first value), or after powering on/switching off tasks exclusion (second value)

Among all ventilators, our reference device the Avea had the better success rate. In between our comparison of the six ventilators, Covidien PB980 had the better results and the Servo-U the worst. A minority of users could power on Servo-U and always over the predefined 120 s time range. Settings reading represented the more difficult task, whatever the device, while their terminology was highly different from one device to the other

* *p* < 0.01 as compared to others. ^§^ *p* < 0.001 as compared to Avea, V500 and PB980. ^¤^ *p* < 0.01 as compared to V680. ^‡^ *p* < 0.05 as compared to others except Avea. ^†^ *p* < 0.05 as compared to PB980 S1 and R860. ^‖^ *p* < 0.001 as compared to Avea and PB980. ^#^ *p* < 0.01 as compared to others, except S1 and V680

#### Psycho-cognitive scales measurements (Figs. 5, 6)

**Fig. 5:**
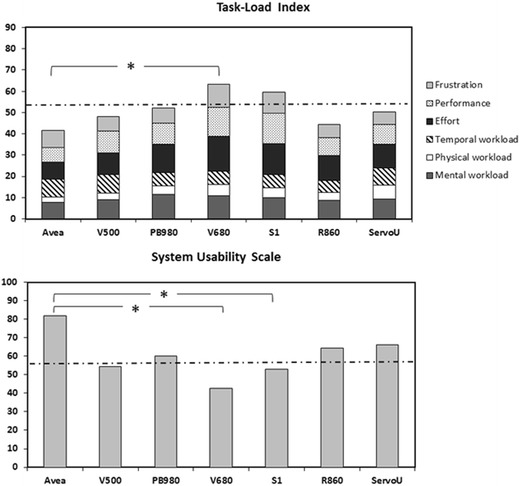
Task Load Index and System Usability Scale scores. *Dotted line* represents the mean value across all scores (Avea excluded). SUS consists of a ten-item questionnaire and assesses usability from different aspects: effectiveness (ability of users to complete tasks); efficiency (level of resource used in performing tasks); and satisfaction (subjective reactions to using the system). SUS score has a range of 0–100, the highest score being the best value (‘simple to use’). NASA-TLX is a multidimensional tool developed for mental workload evaluation. It explores three dimensions dependent on user perception of the task (mental workload, temporal workload and physical workload) and three dimensions dependent on the interaction between the subject and the task itself, which may be mostly related to the interface (effort, performance and frustration). An individual weighting of these dimensions by letting the subjects compare them pairwise enables a decrease in the inter-/intra-individual variability of the overall score. The higher the TLX, the lower the ergonomics. Our reference device (Avea) had the best TLX and SUS scores, and V680 the worst (*p* = 0.049). For usability (SUS), a difference between our reference device (Avea) and S1 was also observed. *p value <0.05

NASA-TLX and SUS scorings are presented in Fig. 5. V680 for NASA-TLX, V680 and S1 for SUS were the only devices to differ significantly from the reference (Avea). Except for the reference, not a single device reached an SUS score equal or higher than 68.

**Fig. 6:**
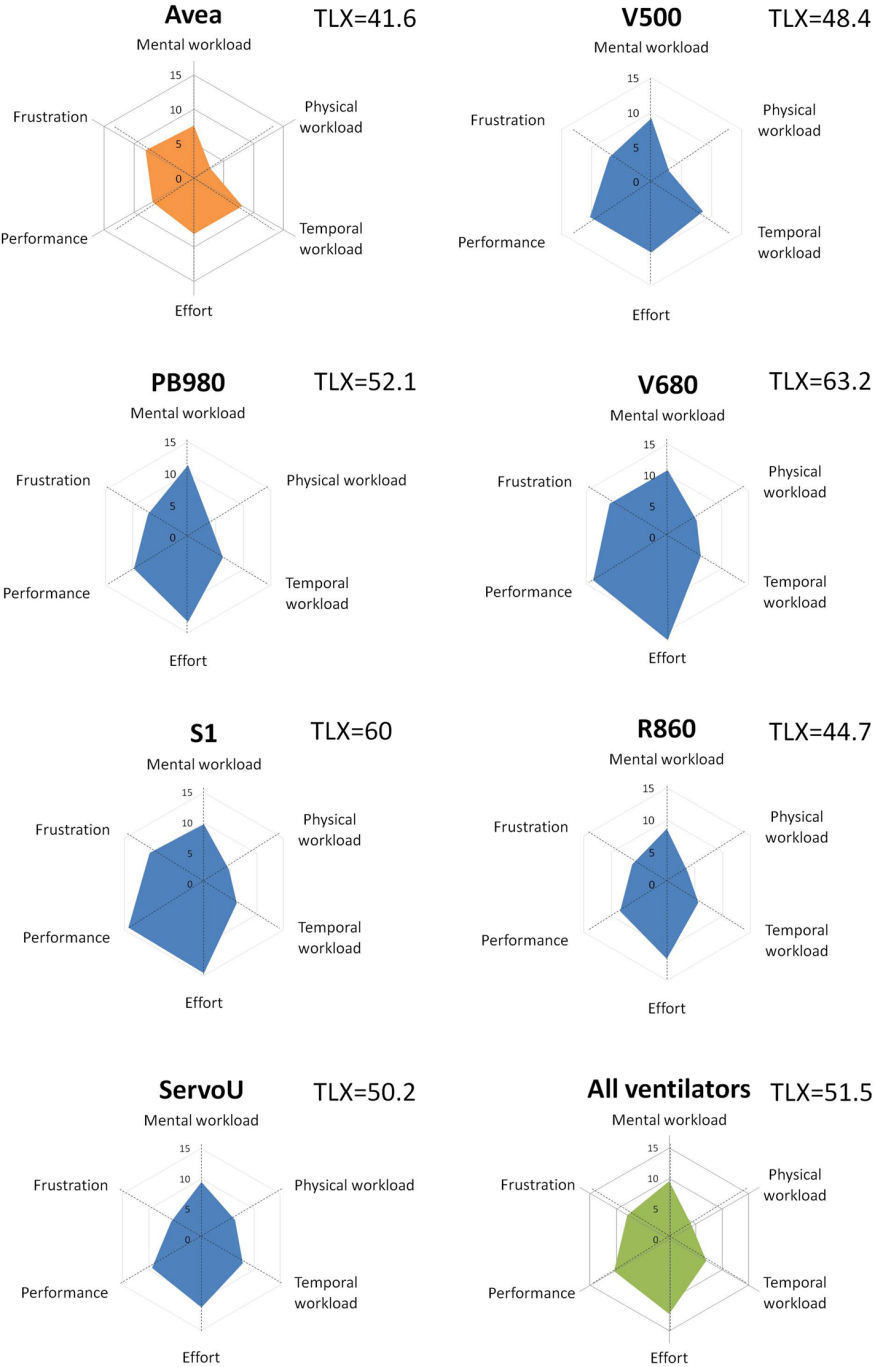
Radar chart of National Aeronautics and Space Administration—Task Load Index for each ventilator. The *radar chart* of the NASA-TLX indicates both the overall mental workload evaluation (TLX value) and the different dimensions that are evaluated. Three dimensions are dependent on user perception of the task (mental workload, temporal workload and physical workload) and three dimensions dependent on the interaction between the subject and the task itself, which may be mostly related to the interface (effort, performance and frustration). The larger the area of the radar chart, the higher the TLX and thus the mental workload, and the lower the ergonomics. Values of the TLX score are indicated for each ventilator, our reference value being depicted in the upper left. Our reference value (Avea, in *orange*) had the lowest mental workload value (TLX = 41.6), thus depicting the potential influence of experience on mental workload. For this reason, it is strictly mandatory to compare measurements performed on naïve subjects. R860 had the lowest TLX value, and V680 had the highest (*p = 0.049). Dimensions of the mental workload that seemed to require the most important improvements were performance and efforts

On the radar chart presentation of the NASA-TLX, except for our reference value (Avea, TLX = 41.6), the R860 had the lowest TLX value (TLX = 44.7) and V680 had the highest (TLX = 63.2; *p* = 0.049). The main dimensions involved in the higher mental workload were ‘performance’ and ‘effort’.

#### Physiological measurements (Fig. 7)

For all parameters, our reference value depicted significantly less activation. Pupillary diameter, respiratory rate and tidal volume activations significantly differed between devices (*p* < 0.05). V500 caused the highest pupillary diameter activation and differed significantly from the reference (*p* = 0.03) and R860 (*p* = 0.019).

**Fig. 7:**
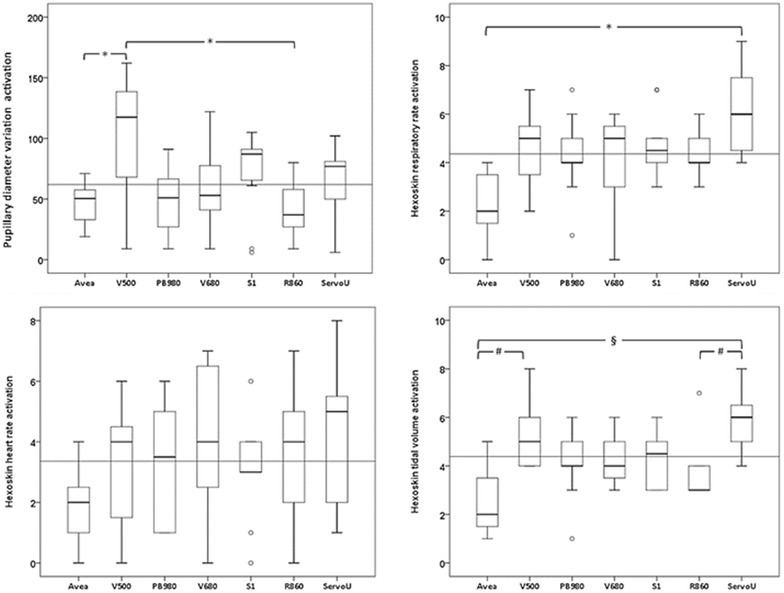
*Box plot* of physiological measurements and eye-tracking activations. Several physiological parameters were recorded during objective tasks completion. These parameters were evaluated while detecting statistically different values, as compared to baseline. Each of these detections (‘*activations*’) is numerically integrated in order to evaluate the number of physiological variations in response to tasks. These activations are considered to be adequate stress indicators. The *number of activations* is represented as median and interquartile. *Dotted line* is mean of activations for all ventilators, during all tests. For all parameters, our reference value depicted significantly fewer activations, thus validating our experimental concept. Significant papillary diameter, respiratory rate and tidal volume activations were observed for several devices (at least V500 and Servo-U). *p value <0.05; ^#^ *p* value <0.005; ^§^ *p* value <0.0005

## Discussion

Within this panel of recently available ICU ventilators, no technical features could be considered as differentiating between devices, while a contrario, ergonomics and interface features were considered inadequate, thus increasing the risks of misusage and adverse events.

### Technical performances

While volume delivery and pressurization accuracy are critical issues, few differences were observed between devices. However, volume delivery and pressurization errors and precisions were important for some ventilators. In all cases, volume delivery was lower than that expected, as already observed [18].

Triggering performances depicted within our study are similar to those observed in a previous study concerning emergency transport ventilators [8] and tended to be higher than previously observed [9, 17]. These results may be explained by different respiratory mechanics and BTPS conditions [8]. No device enabled a triggering delay faster than 50 ms, and it exceeded 100 ms for two devices in normal respiratory mechanics conditions. As already described, flow or pressure triggering has not varied significantly over the last decade [7].

During non-invasive ventilation, patient–ventilator asynchronies are frequent [21, 22] and mainly related to leaks around the interfaces and/or overassistance. Our mean asynchrony index was close to that observed in other studies [8, 23]. Non-invasive ventilation algorithms that are implemented in most devices were able to decrease the asynchrony index significantly and might thus be systematically turned on during non-invasive ventilation, in an attempt to limit non-invasive ventilation failure.

### Ergonomics assessment

While huge effort has been made by manufacturers to improve technical issues, increasing complexity of devices may in fact result in design errors. Not only are the devices’ full capabilities underutilized, but also their main functions may often be handled improperly.

Human error has been demonstrated to be a leading cause of morbidity and death during medical care [10, 24–26]. Many devices have interfaces that are so poorly designed and difficult to use that they can increase the risks associated with the medical equipment and device-induced human error. Human error may be to some extent inevitable and equally caused by human performance and machine performance. In order to limit the number of errors, computing technology and human–machine interface development should be designed to correspond to human characteristics of reasoning and memory constraints [11]. It is also well known that the working memory of humans is limited and that the number of variables depicted on screens is excessive. This results in a large cognitive load (i.e. mental workload) on the user, which is also a determinant of human error [27]. An interface with a human-centred design increases efficiency and satisfaction and decreases the rate of medical error. While these data are integrated in the ventilators’ interface development by manufacturers, and while ergonomics are as essential as technical performances, very few studies have assessed the ergonomics, and many were limited to timed tasks and subjective evaluation [13–15].

To the best of our knowledge, this is first time that such an innovative ergonomics evaluation of ICU mechanical ventilators has been performed, globally integrating the four main dimensions that enable a comprehensive approach to the problem: 1—tolerance to error; 2—ease of use; 3—efficiency; and 4—engagement. Tolerance to error may be directly linked to efficiency and ease of use to engagement. While all four dimensions may be considered independently, they are in fact related one to each other (Fig. 1). Most previous ergonomics evaluations have mainly focused on tolerance to error, while the three other dimensions were often missing data.

The integration of pupil diameter measurement, heart and respiratory rate or tidal volume activation to assess ergonomics are new data in the ICU field. Compared to subjective psychological measurements, these are objective data that allow the estimation of the physiological stress induced by a device’s interface and an indirect assessment of the interface’s usability.

The objective tasks results are often considered as the most representative of the devices’ ergonomics’ differences. Even if we entirely agree with the fact that not all scenarios may have the same importance, it is still surprising that some ventilators could not be powered on by a majority of physicians or that the NIV mode could not be easily activated. Excluding powering on/off tests from analysis, considering that these may be very different tasks that have been voluntarily been made difficult by the manufacturers for safety reasons, did not modify the overall results. While it may be one of the main tasks routinely performed, ventilator setting readings had the worst results of all tasks, probably because of the absence of a homogenized terminology among manufacturers. As already observed in another recent study, the lack of sensitivity of the S1 touch screen was specifically considered by the participants as responsible for an increased mental workload and higher rates of task failures [28]. The physicians praised the Servo-U interface, but the interface also tended to induce high mental workload during specific tasks, thus generating frustration and higher task failure rates.

The pupillary diameter variation is linked to mental workload and is used to assess cognitive skills [29, 30]. However, we must consider the variability related to the light reflex induced by the laboratory environment and the devices themselves [31]. To some extent, this could explain results from the V500 that has a screen luminosity that is higher than that of other devices. Heart and respiratory rates and/or tidal volume variations are linked to emotional behaviour [32–34]. The better results that were observed with the Avea can be explained by the fact that this device was well known to all participants. Our results on the other devices clearly enable the depiction of differences in terms of task completion perceptions among users while using these parameters. Importantly, while the evolution of physiological parameters may not provide comparable results to those obtained with the psycho-cognitive scores, they are consistent with the objective task completion rate results.

The System Usability Scale [20] and NASA Task Load Index [35, 36] are validated psycho-cognitive tools to assess devices’ interface.

The SUS is a very easy scale to administer to participants. It can be used on small sample sizes with reliable results, and it can effectively differentiate between usable and unusable systems. A SUS score above 68 would be considered above average and anything below 68 is considered below average.

The NASA-TLX is a flexible, well-established and widely used multidimensional assessment tool that enables quick and easy workload estimation in order to assess a task or a system. It has been used in a great variety of domains and is considered as one of the most reliable questionnaires to measure workload in a healthcare setting. The higher the weighed TLX, the higher the mental workload and the more ‘difficult to use’ is the device. Each individual dimension can also be considered on its own, either those dependent on users’ perception of the task (mental workload, temporal workload and physical workload) or those dependent on the interaction between the subject and the task itself, which may be mostly related to the interface (effort, performance and frustration). Mental demand describes how much mental and perceptual activity is required to perform the task (e.g. thinking, deciding, calculating). Physical demand describes how much physical activity is required (e.g. pushing, pulling, turning). Temporal demand describes how much time pressure is perceived to fulfil the task (was it slow and leisurely? Or rapid and frantic?). Effort describes how hard the task is to be fulfilled (mentally and physically) in order to accomplish the required level of performance. Performance describes how satisfied the subject feels or whether he/she thinks they were successful in accomplishing the goals. Frustration describes how insecure, discouraged or irritated the subject feels after accomplishing the task. The subscale rating enables inter-/intra-individual variability to be decreased, thus enabling the number of subjects in the experiment to be reduced.

Precedent studies have shown the influence of experience on SUS scores [37], and the better results of the Avea can clearly be related to the users’ knowledge of and experience with this device and not specifically to a better interface. Given the overall expertise of all the physicians from the five ICUs with this device, it was used in the comparison as a reference value.

When considering both psycho-cognitive assessment tools, two devices (V680 and S1) could be considered as below our reference device in terms of usability and induced mental workload. In terms of usability, all devices except the R860 and the Servo-U were equal to or below a SUS value of 60, far below the acceptable average value of 68, which may enable us to consider that from an ergonomics point of view, a huge amount of work has to be done to improve the device’s usability. With regard to the other ventilators, the SUS and NASA-TLX values did not differ, which corresponds with physiological analyses. If devices’ interfaces are globally equivalent, the level of failure observed for some devices, combined with the high induced mental workload and the low usability score, clearly depicts a lack of adaptation of the device’s development to end users. Considering our results and the impact of tasks on dimensions like performance and effort for some devices, manufacturers may primarily focus on interface simplification and rationalization, immediately providing the most important settings and alarms on a first screen, leaving expert settings to a second one. However, given individual physicians’ heterogeneity, the perfect ventilator may be a difficult goal to achieve, and even with experience, some element of frustration and/or temporal workload may still occur, as with our reference device.

### Limitations

As with other bench tests, the main limitations of our study may concern the inability to extrapolate our results to the real clinical situation. First, our technical evaluation was performed on a model, which cannot mimic the complexity of all interactions between a patient and a ventilator. The ASL5000 is a simulator and it remains different from patients, mainly because the spontaneous inspiratory profile is not modified by pressurization during the inspiratory phase. However, the bench simulates most other situations and combinations that can be encountered in the clinical field. Second, the objective and subjective ergonomics measurements were assessed during standardized conditions that may be considered as different from real-life conditions. In order to be able to use various physiological sensors during the ergonomics evaluation, we chose not to use a high-fidelity environment with a manikin. We do agree with the fact that human behaviour under test may be significantly affected by the context and set-up of the experiment. However, while we only included experts, it would have been difficult to reach our experimental goals while also trying to run after a more important degree of immersion that may not be necessary with these types of physicians. A simulated condition may never reproduce all the complexities of the interactions between a patient, a clinician and a ventilator, especially if the tester is an experienced clinician [39]. There are many techniques available for usability evaluation, such as cognitive walk-through, expert reviews, focus groups, Delphi technique, heuristic evaluation or objective timed tasks completion, all of them providing different information [38]. To the best of our knowledge, our study is the only one to provide a global and complete ergonomics evaluation, taking into account different techniques. Third, we may also consider that the small number of senior ICU physicians that were included in our study does not enable firm conclusions to be drawn. Considering the design of the ergonomics evaluation, it required a huge amount of dedicated time from the physicians to undergo the different scenarios and various measurements for the experimental team. Moreover, none of them were familiar with the six tested devices, which exacerbated the difficulty in recruitment. It was therefore unrealistic to use more testers, and such a drawback also tended to be limited by the use of a device that was known to everyone as a comparison and by the fact that we included physicians from five different ICUs. The pairwise comparison that is performed while using the NASA-TLX also limits inter-/intra-individual variability. Finally, the use of the Avea as a ‘reference’ also depicts a specific limitation about the use of subjective psycho-cognitive scales. The better results of the Avea, with both the SUS and the NASA-TLX, clearly indicate that these values may be highly influenced by previous experience. Such a bias was limited within our evaluation by the fact that, in an attempt to assess the ease of use, we only included naive subjects in order to limit the impact of such experience on the evaluation.

## Conclusions

The choice of an ‘*ideal*’ ventilatory device is a difficult task that may concomitantly consider technical performances and ergonomics. While technical bench tests are essential to assess technical performances and a ventilator’s accuracy, a global ergonomics evaluation, taking into account different variables and dimensions, is crucial to enable physicians to focus on their patients, rather than on technological problems. Despite significant technological improvements, several ICU ventilators do exhibit low ergonomics performance and a high risk of misusage.

## Additional file

**Additional file 1: Table S1.** Ventilator’s general characteristics. **Table S2.** Randomisation table for device’s testings. **Table S3.** Participants’ list and cumulated knowledge. **Figure S1.** Pupillar diameter variation measurements. **Figure S2.** Overall inspiratory triggering delay. **Figure S3.** Asynchrony types for each device in the noninvasive ventilation mode.

## Abbreviations

ICU: intensive care unit
NIV: non-invasive ventilation
C: compliance
R: resistance
VT: tidal volume
PEEP: positive end-expiratory pressure
VC-CMV: volume-controlled continuous mandatory ventilation
PC-CSV: pressure-controlled continuous spontaneous ventilation
BTPS: body temperature, pressure and saturated
SUS: System Usability Scale
NASA-TLX: National Aeronautics and Space Administration—Task Load Index
