# Supplementary material for: A new global and comprehensive model for ICU ventilator performances evaluation
Source: Ann Intensive Care. 2017 Jun 17;7:68. doi: 10.1186/s13613-017-0285-2 (PMC5474229; doi:10.1186/s13613-017-0285-2)
Supplement: Supplementary file 1 — Additional file 1: Table S1. Ventilator’s general characteristics. Table S2. Randomisation table for device’s testings. Table S3. Participants’ list and cumulated knowledge. Figure S1. Pupillar diameter variation measurements. Figure S2. Overall inspiratory triggering delay. Figure S3. Asynchrony types for each device in the noninvasive ventilation mode. [file 13613_2017_285_MOESM1_ESM.docx]

**Online Repository**

1. **Additional content - Experimental technical performance evaluation**

All measurements were performed under two levels of FIO_2_ (100%, 50%), three respiratory mechanics combinations (*Normal*: Resistance [R]=5 cmH_2_0.L^-1^.s^-1^; Compliance [C]=70 ml.cmH_2_O^-1^, *Restrictive*: R=10 cmH_2_0.L^-1^.s^-1^; C=30 ml.cmH_2_O^-1^, and *Obstructive:* R=20 cmH_2_0.L^-1^.s^-1^; C=120 ml.cmH_2_O^-1^).

All measures were performed at atmospheric pressure, constant room temperature (22°C) and test-lung temperature (37°C), using an ASL5000™ lung simulator (Ingmar, Pittsburgh, PA, USA). The ASL5000 simulator is a computerized mechanical lung simulator consisting of a piston moving inside a cylinder. The lung model uses the equation of motion to control the movement of the piston. Flow and airway pressure were measured by ﬂow and pressure sensors at the entrance of the piston, and volume was obtained by flow integration over time. The user sets compliance, resistance, and the Pmus waveform. Test-lung temperature was set at the beginning of the experiments, but measurements were performed only after temperature stabilization.

Ventilators were allowed to stabilize in each test condition for 3 to 5 min, after which at least two minutes of ventilation with stable signals were recorded. Data acquisition was performed at 512 Hz and stored in a desktop computer. Offline measurements and curves analysis were performed on a breath-by-breath basis using LabVIEW (National Instruments, Austin, TX, USA) and the dedicated ASL 5000 acquisition software (v3.5).

**I-1 Volume delivery and pressurization accuracy** (see Figure 2)

All measurements were performed under the three respiratory mechanics (normal, obstructive, restrictive), and the two FIO_2_ conditions (50 and 100%).

Volume-controlled-Continuous Mandatory Ventilation reliability was evaluated at VT = 500 mL, RR = 12 b/min, PEEP = 5 and 10 cm H_2_O, without any inspiratory effort.

Pressurization accuracy assessed PEEP (PEEP = 10 cm H_2_O) and inspiratory pressure (pressure support (PS) = 10 cm H_2_O) stability in the pressure support mode (PC-CSV). The intensity of effort was quantified by the pressure decrease at 0.1 s, and three levels of efforts were chosen, as already expressed (P0.1 = 2, 4 and 8 cm H_2_O). A sinusoïdal Pmus waveform was used (Rise time 10%; Inspiratory Hold 10%; Release time 10%). P0.1 was measured using the ASL 5000 sensors. Inspiratory triggering was always set to 2 L/min.

A 10% error value for all these parameters was *a priori* considered clinically relevant for reliability.

**I-2 Triggering evaluation** (see Figure 3)

Experimental conditions reproduced those previously reported and the tested devices were in the same configuration as for PC-CSV evaluation (PS = 10 cm H_2_O, PEEP = 10 cm H_2_O). Simulated patient’s effort intensity was set at P0.1 = 2 cm H_2_O. Inspiratory triggering was always set to flow triggering, at a 2 L/min value.

Triggering performance was assessed according to the following criteria: 1) triggering delay (DT) between onset of the airway pressure decay (beginning of the patient’s effort) and flow delivery (beginning of ventilator pressurization); 2) overall inspiratory delay (DI), which is composed of two components (DT + DP), DP being the pressurization delay, which is defined as the time from the airway pressure signal rise to a return to positive pressure. DI either depends on triggering function and pressurization quality.

**I-3 Asynchrony management**

PSV was delivered at similar respiratory mechanics and P0.1 values, under three levels of circuit leaks ranging from 3.5 to 4.0 (L1), 5.0 to 7.0 (L2) and 9.0 to 12.5 L/minute (L3), using the ASL 5000 exponential leaks module. Two different sets of measurements were performed: 1- using standard PSV settings (invasive mechanical ventilation; no modification of the standard settings, either inspiratory and expiratory trigger values); 2- using specific noninvasive ventilation algorithm.

Leaks were applied using the dedicated ASL5000 Simulator Bypass and Leak Valve Module. This feature can be used to simulate particular patient conditions, such as a face-mask leak under noninvasive ventilation.

Asynchrony index (AI) was calculated over a 1-minute period, after signal stabilization, and took into consideration all major types of asynchrony: ineffective effort, auto-triggering, prolonged inspiration, multiple triggering, premature and short cycling. Ineffective effort was considered when a simulated effort was present on the simulator side, without consecutive inspiration on the ventilator side. Prolonged inspiration was defined as a cycle duration ≥ 50% of a stable cycle without leak.

AI ≥10% of all respiratory efforts was considered clinically significant.

**II- Additional Table S1 – Ventilator’s general characteristics**

| **Device** | **Company** | **Ventilatory Modes** | **FiO2 (range/%)** | **Ventilation Rate** | **Tidal Volume** | **Pressure support** | **PEEP** | **Peak inspiratory flow** | **I : E in VC-CMV** |
| --- | --- | --- | --- | --- | --- | --- | --- | --- | --- |
| **Evita V300** | Dräger (Lubeck, Germany) | VC-CMV, VC-IMV, PC-CMV, PC-IMV, PC-CSV | 100%/21% | 0,5 to 98 /min | 0,1 to 3.0 L | 1 to 95 cmH2O | 0 to 50 cmH2O | 2 to 120 L/min | no specified |
| **PB980** | Newport Covidien (Mansfield, MA, USA) | VC-CMV, VC-IMV, PC-CMV, PC-IMV, PC-CSV | 100%/21% | 0,1 to 100 / min | 0.25 to 2.5 L | 0 to 70 cmH2O | no specified | 3 to 150 L/min | ≤ 1:299 to 4:1 |
| **V680** | Respironics Inc (Murrysville, PA, USA) | VC-CMV, VC-IMV, PC-CMV, PC-IMV, PC-CSV | 100%/21% | 1 to 80 /min | 0.05L to 2.0L | 1 to 65 cmH2O | 0 to 40 cmH2O | no specified | 9.9:1 to 1:9.9 |
| **S1** | Hamilton Medical (Bonaduz, Switzerland) | VC-CMV, VC-IMV, PC-CMV, PC-IMV, PC-CSV | 100%/21% | 5 to 120 /min | 0.100 to 2.0 L | 0 to 100 cmH2O | 0 to 50 cmH2O | 1 to 180 L/min | 1:9 to 4:1 |
| **R860** | General Electric (Fairfield, CT, USA) | VC-CMV, VC-IMV, PC-CMV, PC-IMV, PC-CSV | 100%/21% | 3 to 150 /min | 0,1 to 2L | 1 to 98 cmH2O | 1 to 50 cmH2O | 2 to 160 L/min | 1:9 to 4:1 |
| **Servo U** | Maquet Getinge (Göteborg, Sweden) | VC-CMV, VC-IMV, PC-CMV, PC-IMV, PC-CSV | 100%/21% | 4 to 100 /min | 0.1 to 4.0L | 0 to 120 cmH2O | 1 to 50 cmH2O | 0.5 to 60 l/min | 1:10 to 4:1 |
| **Avea** | Viasys Carefusion (San Diego, Ca, USA) | VC-CMV, VC-IMV, PC-CMV, PC-IMV, PC-CSV | 100%/21% | 1 to 120 /min | 0.002 to 2.5L | 0 to 90 cmH2O | 0 to 50 cmH2O | 0.4 to 150 L/min | no specified |

1. **Additional content – Ergonomics evaluation**

**Additional Table S2:** Randomisation table for device’s testings

| ***Participants Number*** | ***Ventilator type*** | | | |
| --- | --- | --- | --- | --- |
| 1 | Avea | Covidien | Hamilton | GE |
| 2 | Avea | Covidien | Hamilton | Maquet |
| 3 | V500 | Covidien | Hamilton | Maquet |
| 4 | Covidien | Hamilton | GE | Maquet |
| 5 | Avea | V500 | Hamilton | Maquet |
| 6 | Avea | V500 | Covidien | GE |
| 7 | Avea | V500 | GE |  |
| 8 | Avea | Covidien | Philips | GE |
| 9 | V500 | Philips | GE | Maquet |
| 10 | V500 | Philips | Hamilton | Maquet |
| 11 | V500 | Covidien | Philips | Maquet |
| 12 | V500 | Philips | Hamilton | GE |
| 13 | Avea | Philips | GE | Maquet |
| 14 | Avea | Covidien | Philips | Hamilton |
| 15 | Avea | V500 | Covidien | GE |
| 16 | Avea | Philips | GE | Maquet |
| 17 | Avea | Covidien | Philips | Hamilton |
| 18 | V500 | Covidien | Philips | Maquet |
| 19 | V500 | Philips | Hamilton | GE |
| 20 | Avea | V500 | Hamilton | Maquet |

*This table details the randomization table for devices’ testings by the ICU physicians. All were already experienced with the Avea but still performed testing on this device, as a reference. They were randomly assigned, according to their previous experience, for the testing of 2 to 3 other devices.*

**Additional Table S3:** Participants’ list and cumulated knowledge

| **Tested Ventilators** | **Participants** | **Cumulated knowledge**  n (%) |
| --- | --- | --- |
| Avea | 1, 3, 5, 6, 7, 8, 13, 14, 15, 16, 17, 20 | 20 (100) |
| S1 | 1, 2, 3, 4, 5, 10, 12, 14, 17, 19, 20 | 5 (25) |
| V500 | 3, 5, 6, 7, 9, 10, 11, 12, 15, 18, 19, 20 | 4 (20) |
| PB980 | 1, 2, 3, 4, 6, 8, 11, 14, 15, 17, 18 | 1 (5) |
| V680 | 8, 9, 10, 11, 12, 13, 14, 16, 17, 18, 19 | 0 (0) |
| R860 | 1, 4, 6, 7, 8, 9, 12, 13, 15, 16, 19 | 0 (0) |
| Servo U | 2, 3, 4, 5, 9, 10, 11, 13, 16, 18, 20 | 0 (0) |

*This table depicts previous knowledge on other devices from the same manufacturer. Except from the Avea that was known by all physicians, none had already used in clinical routine the device that they were testing. However, a few of them had already used other devices from the same manufacturer.*

For ergonomics evaluation, we included as a reference, the use of a device that was well known by all physicians (Avea, Carefusion – Becton Dickinson, Franklin Lakes, NJ, USA).

20 senior ICU physicians from 5 different ICUs were included in the evaluation. Each physician tested from three to four devices, in a randomized order; each device was tested from eleven to twelve times. All physicians were familiar with the Avea (Carefusion, San Diego, Ca, USA), that was used as a reference; we took particular attention to the fact that none of them were familiar with the tested devices (“naive” subjects), even if some of them were a few times be familiar with other devices from the same manufacturer (see Online Repository – Table 2).

1. **Additional figures legends**

**Additional Figure S1:** Pupillar diameter variation measurements

Pupil diameter was assessed using an eye-tracking system (SMI ETG 1, SensoMotoric Instruments GmbH, Teltow, Germany). Parameters variations were automatically assessed using SMI Experiment Suite Scientific software.

In addition to its reactivity to light intensity, the diameter of the pupil is affected by many parameters which are unrelated to visual stimulation, such as emotions, target detection, decision making, and others. However, the most widely recognized cognitive factor influencing pupil size is mental load. For such reasons, pupil diameter modifications were integrated as physiological parameters in our bench test.

**Additional Figure S2:** Overall inspiratory triggering delay

Values are provided as mean + SD. Black box represents DT: triggering delay, from the onset of the airway pressure decay (beginning of the patient’s effort) to flow delivery (beginning of ventilator pressurization); White box represents DP: pressurization delay, from the airway pressure signal rise to a return to positive pressure; DI is the overall inspiratory triggering delay (DT + DP).

No differences could be observed in terms of inspiratory triggering delay in between devices.

**Additional Figure S3:** Asynchrony types for each device in the noninvasive ventilation mode

Remaining asynchrony types were evaluated for each device under the NIV mode. Two devices depicted an AI below 10% under the NIV mode (R860 and Servo U). Most frequent asynchronies were prolonged cycles and ineffective efforts (IE), IE being most of the time associated with prolonged cycles.

* P<0.05
